# Systematic review and meta-analysis of modified facelift incision versus modified Blair incision in parotidectomy

**DOI:** 10.1038/s41598-021-03483-6

**Published:** 2021-12-16

**Authors:** Yi-Chan Lee, Wei-Chih Liao, Shih-Wei Yang, Cheng-Ming Luo, Yao-Te Tsai, Ming-Shao Tsai, Yi-Hsuan Lee, Li-Jen Hsin

**Affiliations:** 1 Department of Otolaryngology-Head and Neck Surgery, Chang Gung Memorial Hospital, Keelung, Taiwan; 2 Department of Otolaryngology-Head and Neck Surgery, Chang Gung Memorial Hospital, No. 5 Fushing St., Taoyuan, 333 Taiwan; 3 Department of Otolaryngology-Head and Neck Surgery, Chang Gung Memorial Hospital, Chiayi, Taiwan; 4 College of Medicine, Chang Gung University, Taoyuan, Taiwan; 5 Department of Orthopedic Surgery, Buddhist Tzu-Chi General Hospital, Taipei, Taiwan

**Keywords:** Anatomy, Medical research, Oncology

## Abstract

Surgical removal is the treatment of choice for many neoplasms of the parotid gland. This meta-analysis aimed to evaluate the differences between parotidectomy using a modified facelift incision (MFI) and parotidectomy using a modified Blair incision (MBI). A systematic search of the available literature in PubMed, Embase and the Cochrane Library was performed. Studies of adult patients who underwent open parotidectomy with presumed benign parotid neoplasms based on preoperative examinations were reviewed. The surgical outcomes of the MFI and MBI groups were collected. Intraoperative and postoperative parameters, including operative time, tumor size, cosmetic satisfaction, and incidences of facial palsy, Frey’s syndrome and salivary complications, were compared. Dichotomous data and continuous data were analyzed by calculating the risk difference (RD) and the mean difference (MD) with the 95% confidence interval (CI), respectively. Seven studies were included in the final analysis. The pooled analysis demonstrated that the cosmetic satisfaction score was significantly higher in the MFI group (MD = 1.66; 95% CI 0.87–2.46). The operative duration in the MFI group was significantly longer than that in the MBI group (MD = 0.07; 95% CI 0.00–0.14). The MFI group exhibited a smaller tumor size (MD = − 2.27; 95% CI − 4.25 to − 0.30) and a lower incidence of Frey’s syndrome (RD = − 0.18; 95% CI − 0.27 to − 0.10). The incidence of postoperative temporary facial palsy (RD = − 0.05; 95% CI − 0.12 to 0.03), permanent facial palsy (RD = − 0.01; 95% CI − 0.06 to 0.03) and salivary complications (RD = − 0.00; 95% CI − 0.05 to 0.05) was comparable between the two groups. Based on these results, MFI may be a feasible technique for improving the cosmetic results of patients who need parotidectomy when oncological safety can be ensured.

## Introduction

Salivary gland tumors account for less than 3% of all neoplasms. Approximately 80% of these tumors are of parotid origin, and of these tumors, 80% are benign^1^. Surgical removal is the treatment of choice for most neoplasms of the parotid gland.

The classic cervicomastoidfacial incision for parotidectomies was introduced by Blair in 1912^2^. Bailey then proposed the modified Blair incision (MBI) in 1941; the MBI omits the part of the incision that runs parallel to the zygomatic arch^3^. Since then, the MBI has become the most commonly used conventional incision for parotidectomies. MBI is able to provide excellent surgical exposure to the parotid gland but leaves a visible cervical scar after surgery, leading to cosmetic dissatisfaction in some patients. Several surgical techniques have been proposed in the pursuit of better cosmesis after parotidectomy^4–6^.

The incision designed for facelift surgery, also known as rhytidectomy, is one of the approaches considered for this purpose. The traditional facelift incision consists of a preauricular segment extending to the temporal scalp and a retroauricular segment extending horizontally to the hair-bearing scalp. In the literature, however, most authors preferred using the modified facelift incision (MFI) in parotid surgeries. The MFI differs from the traditional facelift incision in that there is no temporal scalp incision, and the retroauricular incision continues inferiorly along the hairline rather than horizontally. With the use of MFI, the cervical incision needed for surgery is moved further back into the hairline; thus, a visible cervical scar is avoided.

Cosmetic satisfaction after parotidectomy may be improved by using the MFI, and several authors have reported the aesthetic superiority of MFI. However, to date, no meta-analysis has been published that evaluates the difference between these two techniques. In the present study, the authors conducted a systemic review of related articles and presented the combined results of the postoperative and intraoperative parameters after the use of MFI and MBI in parotidectomy.

## Materials and methods

### Literature search

This meta-analysis was performed in accordance with the Preferred Reporting Items for Systematic Reviews and Meta-Analyses statement^7^. Two authors (LJH and YCL) extensively and independently searched PubMed, Embase, and the Cochrane Library for studies of interest published before December 2020. The keywords in our search process included “parotid”, “parotidectomy”, “facelift”, “rhytidectomy”, “cosmetic” and “esthetic”. Moreover, the references of the included articles were also reviewed to identify other potential studies.

### Study selection

The PICO (population/intervention/comparison/outcome) components were as follows: P (adult patients who underwent open parotidectomy with presumed benign parotid neoplasms based on preoperative examinations), I (use of MFI in parotidectomy), C (use of MBI in parotidectomy), O (intraoperative and postoperative parameters, including operative time, tumor size, cosmetic satisfaction, incidences of facial palsy, Frey’s syndrome and salivary complications).

The inclusion criteria were as follows: (1) original research articles with either prospective or retrospective study design; (2) articles published in the English language; (3) articles that included adult patients who underwent open parotidectomy with presumed benign parotid neoplasms based on preoperative examinations; (4) studies comparing intraoperative and postoperative outcomes between the MFI and MBI groups; and (5) studies including follow-up of at least 3 months after surgery. The exclusion criteria were as follows: (1) studies that included patients with known parotid malignancies before surgery; (2) studies using endoscope-assisted or robot-assisted surgery; (3) studies using a fibrin sealant after parotid surgery; (4) studies with flap or fascia reconstruction after parotidectomy; (5) studies without a control group; (6) articles not published in English; and (7) review articles, short reports, letters to the editor and cadaveric studies.

The MFI described in the present study includes a preauricular incision that extends around the origin of the earlobe, following the retroauricular sulcus. The incision then curves toward the occipital direction and can be continued with a segment along the hairline as needed. The temporal scalp segment and horizontal segment over the occipital scalp in traditional facelift incision are not included in the MFI. The MBI, on the other hand, starts in the preauricular skin crease, continues posteriorly around the lobule to the mastoid region, and extends inferiorly into a cervical skin crease. The segment running parallel to the zygomatic arch used in the original Blair incision is not included in the MBI used in the present study (eFigure 1).

### Data extraction

Data were independently extracted by 2 researchers (LJH and YCL). The quality of the included articles was independently assessed by two researchers (LJH and YCL) using the Newcastle–Ottawa Scale^8^. Any discrepancies in the study bias classification were resolved by discussion among authors until a consensus was achieved.

### Outcomes

The outcomes of this meta-analysis included cosmetic satisfaction, operative duration (hours), tumor size (centimeters), and incidence of postoperative facial palsy, Frey’s syndrome and salivary complications.

### Data analysis

The results of interest were analyzed with Comprehensive Meta-Analysis software (version 3; Biostat, Englewood, NJ). The mean difference (MD) was used to compare the cosmetic satisfaction score, operative duration and tumor size between the MFI and MBI groups. The risk difference (RD) was used to compare the incidence of postoperative facial palsy, Frey’s syndrome and salivary complications between the MFI and MBI groups. When necessary, the mean and standard deviation were estimated according to the methods described in previous studies^9,10^. The overall effect was calculated using a random-effects model. Grading of Recommendations, Assessment, Development and Evaluations (GRADE) was used to assess the quality of the evidence for each outcome^11^. Heterogeneity among studies was analyzed using the *I*^2^ statistic, which calculated the proportion of overall variation attributable to between-study heterogeneity. An *I*^2^ value exceeding 50% suggested moderate heterogeneity, and an *I*^2^ value exceeding 75% suggested high heterogeneity^12^. Potential publication bias was analyzed using the Egger intercept test and funnel plots when more than 10 studies were present per outcome^13^. A 2-tailed *P* value of less than 0.05 was considered statistically significant.

## Results

### Study selection

**Figure 1:**
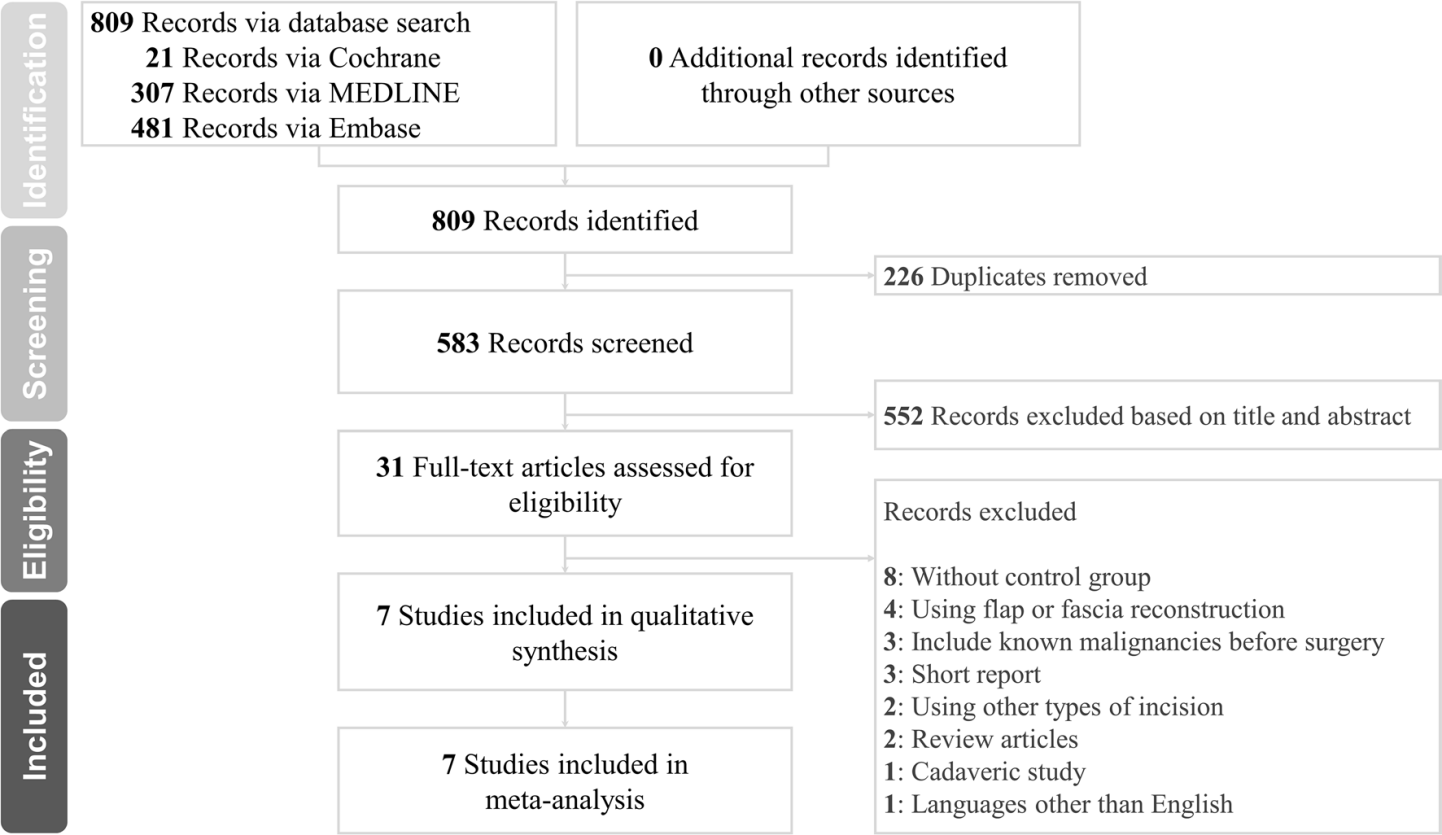
Flow diagram of the literature search.

The literature search initially yielded 809 articles. A total of 226 duplicate studies were excluded; 552 studies were excluded after reviewing the titles and abstracts. Careful review of the full text was performed for the remaining 31 potentially eligible articles. Among these articles, studies without a control group, review articles, studies including fascia or flap reconstruction, short reports, studies using incisions other than the MFI, studies including known parotid cancer patients before surgery, cadaveric studies and studies not published in the English language were excluded. Seven articles were included in the final analysis^14–20^. A flow diagram describing the process of study selection and inclusion/exclusion is shown in Fig. 1. The keywords used and the literature search are described in eTable 1 of the Supplementary material.

### Demographics

The basic demographics of the included study subjects are listed in Table 1. A total of 707 parotidectomies were included for analysis. The overall male/female ratio was significantly lower in the MFI group than in the MBI group (*P* < 0.00). The PRISMA checklist can be found in the eTable 2 of the Supplementary material. The quality assessment for the included studies is shown in eTable 3 of the Supplementary material.

**Table 1:** Characteristics of the included studies.

Author	Year	Country	Study design	Follow-up (months)	Surgery type	Final diagnosis	Number of parotidectomies		Mean age (years)		Sex (M/F)	
							MFI	MBI	MFI	MBI	MFI	MBI
Terris et al.	1994	US	Retrospective	7.9^a^	S/T	PA/ACC/MC/AC	18	15	40.3	40.3	1/16	5/10
Wasson et al.	2010	UK	Retrospective	≥ 6	Pa	NA	20	59	44.0	51.0	11/9	29/30
Bianchi et al.	2011	Spain	Retrospective	≥ 18	Pa	PA/WT	48	35	NA	NA	NA	NA
Lee et al.	2011	Korea	Retrospective	≥ 6	S/Pa/ST	PA/WT/BA/LN	182	162	44.1	45.8	51/131	90/72
Graciano et al.	2013	Brazil	Retrospective	≥ 6	Pa	NA	30	30	34.9	47.3	11/19	21/9
Kim et al.	2014	Korea	Retrospective	29^b^	PSP	PA/WT/BA/CX/MC	24	16	49.8	45.3	9/15	6/10
Zhang et al.	2019	China	Retrospective	≥ 6	PSP	PA/WT/ME/EA/LC	32	36	NA	NA	NA	23/13
											83/190	174/144
											*P* < 0.00^c^	

*M* male, *F* female, *MFI* modified facelift incision, *MBI* modified Blair incision, *US* United States, *HK* Hong Kong, *UK* United Kingdom, *S* superficial parotidectomy, *T* total parotidectomy, *Pa* partial parotidectomy, *ST* subtotal parotidectomy, *PSP* partial superficial parotidectomy, *NA* not available, *PA* pleomorphic adenoma, *ACC* adenoid cystic carcinoma, *MC* mucoepidermoid carcinoma, *AC* acinic cell carcinoma, *WT* Warthin’s tumor, *BA* basal cell adenoma, *LN* lymph node hyperplasia, *CX* carcinoma ex pleomorphic adenoma, *ME* myoepithelioma, *EA* eosinophilic adenoma, *LC* lymphoepithelial cyst.

^a^Follow-up period is presented as the mean.

^b^Follow-up period is presented as the median.

^c^*P*-value from the chi-square test comparing the distribution of males and females in the MFI and MBI groups.

### Outcomes

#### Cosmetic satisfaction

**Figure 2:**
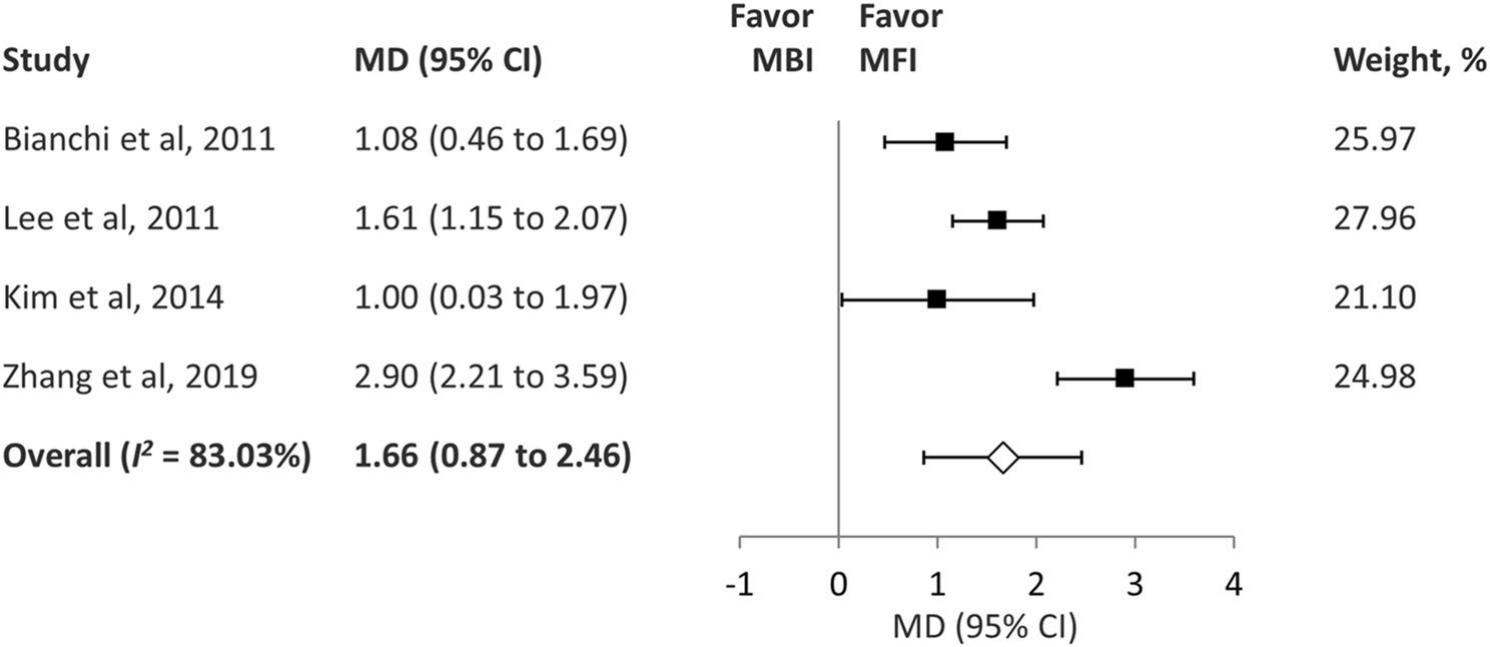
Forest plot of cosmetic satisfaction after parotidectomy. *MD* mean difference, *CI* confidence interval, *MBI* Blair incision, *MFI* modified facelift incision.

Five of the studies recorded cosmetic satisfaction with a numerical scale, on which 10 points indicated the highest score of cosmetic satisfaction^14,16,17,19,20^. Four articles provided sufficient data for analysis^16,17,19,20^. One study evaluated the cosmetic results 3–4 months after surgery^19^, two studies evaluated the cosmetic results at least 6 months after surgery^17,20^, and one study evaluated the cosmetic results at least 18 months after surgery^16^. The pooled analysis of the four studies demonstrated that the cosmetic satisfaction score was higher in the MFI group than in the MBI group (MD, 1.66; 95% confidence interval [CI] 0.87–2.46; *I*^2^ = 83.03%) (Fig. 2). We did not perform subgroup analysis due to the number of eligible studies. However, the study reported by Zheng et al. was found to be the source of heterogeneity^20^. The heterogeneity was obviously reduced after this study was removed from the analysis (*I*^2^ = 19.84%). The study by this group attempted to shorten the hairline segment of the MFI and only extended this segment along the hairline when necessary. Other authors indicated that they regularly used the hairline limb of the MFI along the hairline, which might cause heterogeneity between studies. GRADE indicated evidence of moderate quality for this outcome (eTable 4).

#### Operative duration

**Figure 3:**
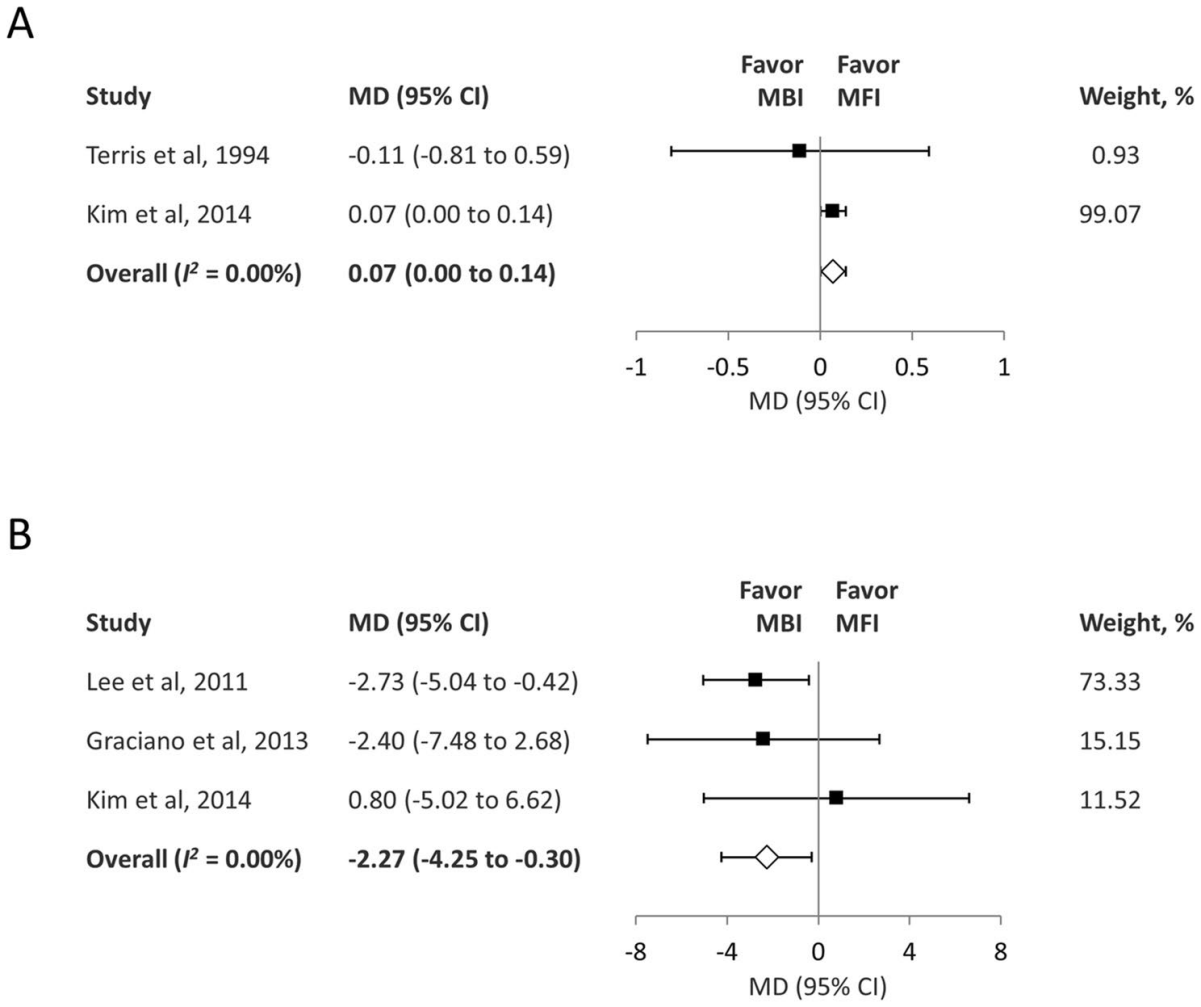
(**A**) Forest plot of the operative time during parotidectomy (hours). (**B**) Forest plot of the tumor size (centimeters). *MD* mean difference, *CI* confidence interval, *MBI* Blair incision, *MFI* modified facelift incision.

The pooled analysis of two studies^14,19^ showed that the operative duration was lower in the MBI group than in the MFI group (MD = 0.07; 95% CI 0.00–0.14; *I*^2 ^= 0.00%) (Fig. 3A). GRADE indicated evidence of moderate quality for this outcome (eTable 4).

#### Tumor size

The pooled analysis of three studies^17–19^ showed that the tumor size was smaller in the MFI group than in the MBI group (MD = − 2.27; 95% CI − 4.25 to − 0.30; *I*^2 ^= 0.00%) (Fig. 3B). GRADE indicated evidence of moderate quality for this outcome (eTable 4).

#### Postoperative facial palsy

Four studies recorded the incidence of temporary facial palsy^16,18–20^, and five studies recorded the incidence of permanent facial palsy^14,16,18–20^. Meta-analyses were performed for both temporary and permanent facial palsy.

**Figure 4:**
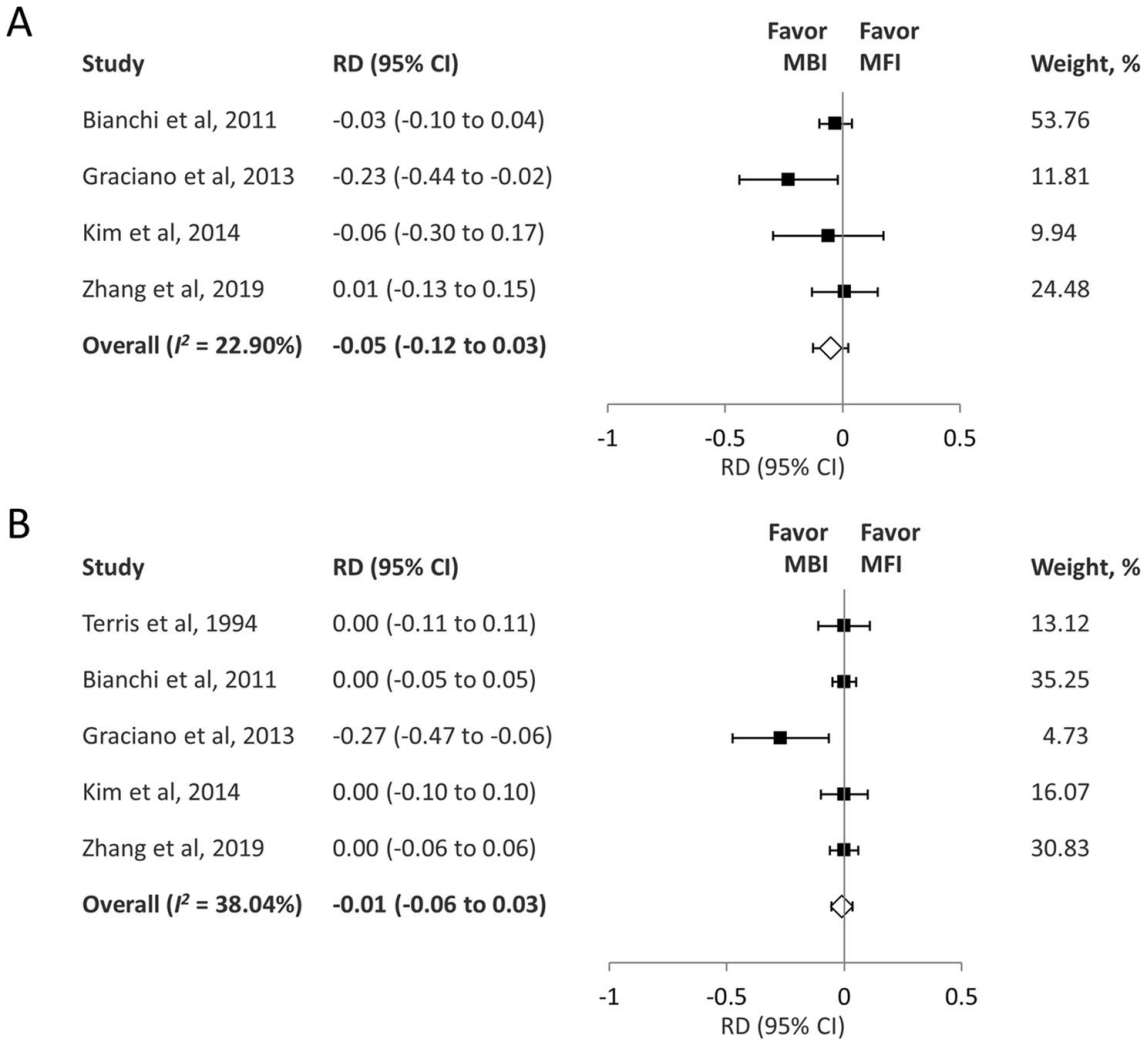
(**A**) Forest plot of the incidence of temporary facial palsy after parotidectomy. (**B**) Forest plot of the incidence of permanent facial palsy after parotidectomy. *RD* risk difference, *CI* confidence interval, *MBI* Blair incision, *MFI* modified facelift incision.

The pooled analysis demonstrated that the rate of temporary facial palsy was comparable between the two groups (RD − 0.05; 95% CI − 0.12 to 0.03; *I*^2 ^= 22.90%) (Fig. 4A). The pooled analysis demonstrated that the rate of permanent facial palsy was comparable between the two groups (RD − 0.01; 95% CI − 0.06 to 0.03; *I*^2 ^= 38.04%) (Fig. 4B). GRADE indicated evidence of moderate quality for this outcome (eTable 4).

#### Postoperative Frey’s syndrome

**Figure 5:**
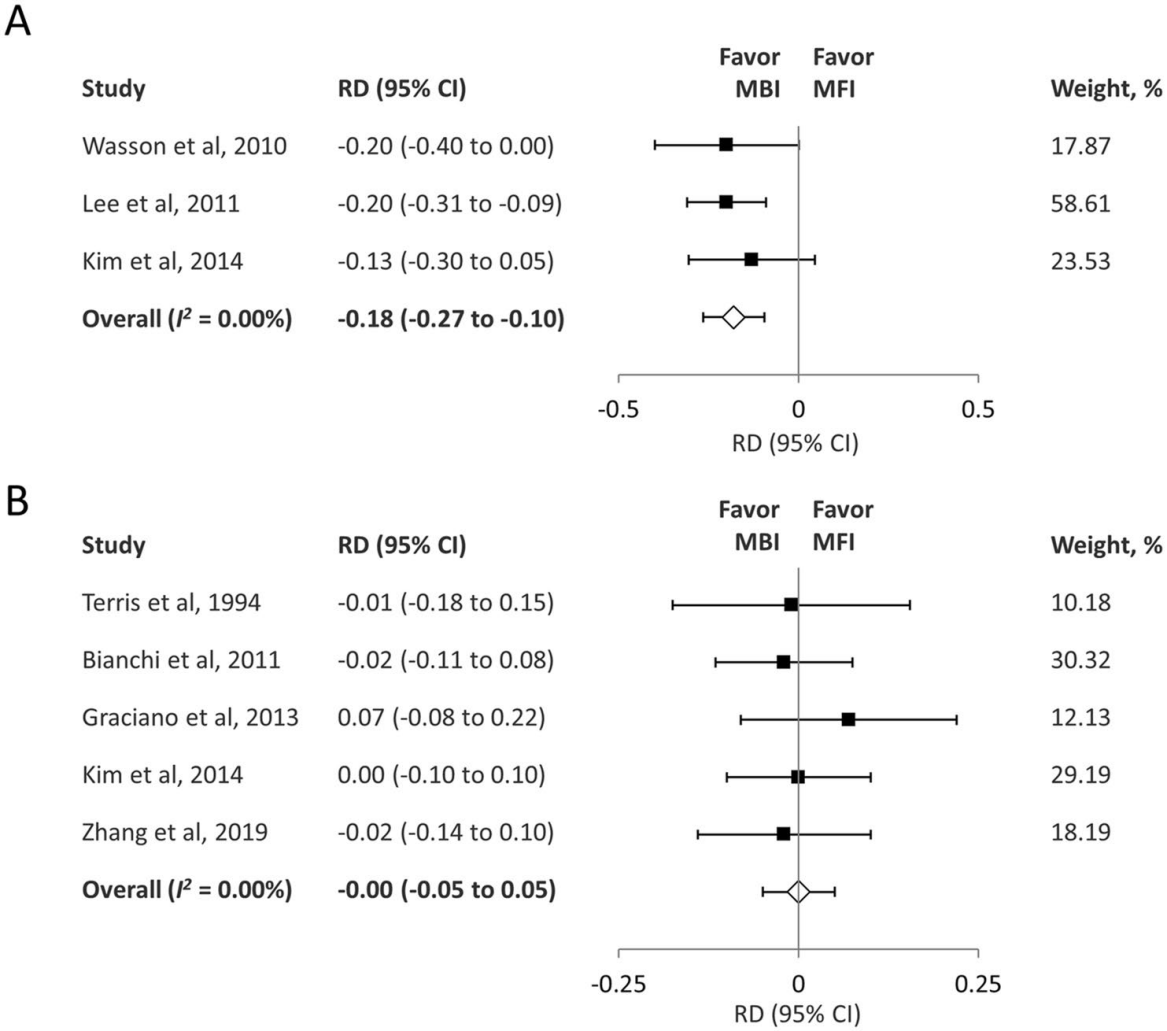
(**A**) Forest plot of the incidence of Frey’s syndrome after parotidectomy. (**B**) Forest plot of the incidence of salivary complications after parotidectomy. *RD* risk difference, *CI* confidence interval, *MBI* Blair incision, *MFI* modified facelift incision.

The pooled results of three studies^15,17,19^ showed that the rate of postoperative Frey’s syndrome was lower in the MFI group than in the MBI group (RD = − 0.18; 95% CI − 0.27 to − 0.10; *I*^2^ = 0.00%) (Fig. 5A). GRADE indicated evidence of moderate quality for this outcome (eTable 4).

#### Salivary complications (salivary fistula/seroma)

The pooled results of five studies^14,16,18–20^ showed no significant difference between the two groups in terms of the rate of salivary complications (RD = − 0.00; 95% CI − 0.05 to 0.05; *I*^2^ = 0.00%) (Fig. 5B). GRADE indicated evidence of moderate quality for this outcome (eTable 4).

### Publication bias

The funnel plots were under powered when fewer than 10 studies were included in the meta‐analysis according to the recommendations of the Cochrane handbook^13^. As only seven studies were included in this review, publication bias was not evaluated.

## Discussion

This meta-analysis of the existing English literature was performed to compare the differences between the MFI and MBI techniques in parotidectomy. One study in 2013 published a systematic review regarding parotid surgeries using the MFI; however, the authors did not perform meta-analyses, probably due to the lack of sufficient data^21^. The present study, which included seven studies and 707 parotidectomies, is the first meta-analysis comparing parameters between the MFI and MBI. According to the pooled results, the MFI group reported a significantly higher cosmetic satisfaction score than the MBI group. Compared with those who underwent MBI parotidectomy, patients who underwent MFI parotidectomy had a longer operative duration, a smaller tumor size, and a decrease in the incidence of postoperative Frey’s syndrome. The incidence of postoperative salivary complications, temporary and permanent facial palsy was comparable between the two groups.

The fundamental function of an incision is to provide adequate surgical field exposure and lesion access. With the advancement and development of surgical techniques, physicians have begun to explore possible incisions with satisfactory cosmetic outcomes without jeopardizing oncologic safety. The conventional incision described by Blair in 1912, with its modifications, has been well studied and established as the most utilized approach in parotid surgeries^2^. However, an obvious scar on the face and neck after using the MBI is unavoidable, even with meticulous closure. According to previous studies regarding parotid surgeries, long-term evaluation by questionnaires seems to indicate high scar dissatisfaction^22,23^. Some authors have even reported that scars represent the most important long-term issue^22^. Several types of incisions have been proposed to improve aesthetic results after parotid surgery, and the MFI may be the most widely used technique. With the use of the MFI in parotidectomy, the scar is hidden behind the tragus, retroauricular sulcus and natural hairline. The postoperative scar is inconspicuous or visible only under close inspection. Five of the included articles in the present meta-analysis recorded cosmetic results after surgery. Among these studies, the study by Wasson et al. was the only one to report a higher mean cosmetic satisfaction score in the MBI group than in the MFI group. However, the authors did not provide a statistical comparison between the two groups; thus, the study was not included in our pooled analysis^13^. The other four studies all showed a higher satisfaction score in the MFI group than in the MBI group. The pooled results indicated that the use of the MFI significantly improved the cosmetic satisfaction score after parotidectomy.

The results of the present study show that the operative duration for parotidectomy is approximately 4.2 min longer with the use of the MFI than with the use of the MBI. This result is intuitive because the use of MFI requires a greater extent of flap dissection to expose the surgical field. However, some authors have indicated that the time needed for flap dissection may decrease as the surgeon becomes more experienced. Other factors, such as the surgery type, tumor size or location, may also be associated with the operation duration^24^. The present study also showed that the parotid tumor size was smaller in the MFI group than in the MBI group. Although different medical facilities may have different protocols for choosing the incision type and comparable tumor sizes between the two incision groups have been reported^18,19^, the presented pooled data suggest that physicians seem to consider using the MFI for patients with smaller tumors. One of the included studies suggested that MFI is more suitable for tumors in the lower and posterior portions of the parotid gland^20^, while several authors indicated that MFI can be used in parotidectomy regardless of tumor location^17,19^. However, the information regarding tumor location is limited and not reported with a universal method, which makes pooled analysis difficult. Future studies focusing on the relationship between tumor location and incision type may be useful.

Facial palsy leads to both functional impairments and esthetic complaints, negatively affecting the quality of life of patients. Preservation of facial nerve function is therefore one of the most critical surgical steps in parotidectomy. In the present meta-analysis, both the incidence rates of temporary and permanent facial palsy were comparable between the two groups. One cadaveric study from 2010, which compared the achieved surgical field with the MFI and MBI approaches, also revealed no significant difference in the extent of exposure^25^. The use of electromyography in intraoperative facial nerve monitoring was introduced in 1970, and it has become increasingly popular in recent years. One review article from 2020 suggested that the risk of temporary and permanent facial nerve weakness after primary parotid gland surgery may be decreased with the use of a nerve monitoring system^26^. However, only one of the articles included in this study described the use of a nerve monitoring system in the surgical process^18^. The proper use of a nerve monitoring system may not only help to protect facial nerve function but also potentially decrease the time needed for surgery during parotidectomy with the MFI approach.

Frey’s syndrome is caused by anatomical communication between the sweat glands of the face and the severed postganglionic parasympathetic nerve fibers supplying the parotid gland. Three of the included studies recorded the incidence of Frey’s syndrome after surgery. However, none of these studies described the use of objective methods such as Minor’s test in the diagnosis of Frey’s syndrome. The true incidence may therefore be higher than reported if objective examinations are used. The pooled results in our analysis demonstrated that the incidence rate of Frey’s syndrome was lower in the MFI group than in the MBI group. Tumor size has been considered a significant predictor of Frey’s syndrome after parotidectomy^27^. The tumor size in the present study was smaller in the MFI group than in the MBI group, which may partly explain the lower incidence of Frey’s syndrome in the MFI group. Other factors, such as the extent of surgery and the histological type of the tumor, have also been reported to be associated with the occurrence of Frey’s syndrome^28^.

A sialocele is defined as the accumulation of saliva in the parotid region after parotidectomy, and a salivary fistula can occur if these fluid collections drain onto the skin. Our results showed that there was no significant difference in the incidence rate of salivary complications between the MFI and MBI groups.

The authors acknowledge the limitations of this study. First, we included only 7 retrospective studies in this meta-analysis. More well-controlled studies may be required to further confirm these results. Second, the types of parotidectomy, the size of tumors, the time of follow-up and the time to assess cosmetic satisfaction may all have potential influences on the outcomes of interest, and these results need to be interpreted with caution. Third, we were not able to analyze the recurrence rate because only three of the included studies provided the data regarding tumor recurrence, and the follow-up period was 18 months at most. Parotid tumors are reported to recur between 4.7 and 9.1 years, which exceeds the follow-up periods of these studies^29–31^. Despite these limitations, the present meta-analysis still provides evidence for the use of different incisions in parotidectomy.

## Conclusion

In conclusion, the patients who underwent parotidectomy with the MFI demonstrated a significantly higher cosmetic satisfaction score than those who underwent parotidectomy with the MBI. In our study, compared with the use of the MBI in parotidectomy, the use of the MFI in parotidectomy was associated with a longer operative duration, a smaller tumor size, and a decrease in the incidence of postoperative Frey’s syndrome. In addition, we observed a similar rate of postoperative salivary complications, temporary and permanent facial palsy between the MFI and MBI groups. Optimal local disease control is still the primary aim of surgical intervention in parotid tumors. Physicians may consider using the MFI for patients with particular cosmetic concerns, such as younger patients, when oncological safety can be ensured.

## Supplementary Information

Supplementary Information.
