# Supplementary material for: Systematic review and meta-analysis of modified facelift incision versus modified Blair incision in parotidectomy
Source: Sci Rep. 2021 Dec 16;11:24106. doi: 10.1038/s41598-021-03483-6 (PMC8677756; doi:10.1038/s41598-021-03483-6)

**Supplementary Online Content**

**eTable 1.** Literature Searches and Keywords

| - **Medline**   (facelift or rhytidectomy or cosmetic or esthetic or aesthetic or retroauricular or postauricular) AND (parotidectomy) |
| --- |
| - **Cochrane**   (facelift or rhytidectomy or cosmetic or esthetic or aesthetic or retroauricular or postauricular) in Title Abstract Keyword AND (parotidectomy) in Title Abstract Keyword - (Word variations have been searched) |
| - **Embase**   (facelift OR 'rhytidectomy'/exp OR rhytidectomy OR 'cosmetic'/exp OR cosmetic OR esthetic OR aesthetic OR retroauricular OR postauricular) AND ('parotidectomy'/exp OR parotidectomy) |

**eTable 2:** PRISMA checklist

| **Section and topic** | **Item #** | **Checklist item** | **Reported on page #** |
| --- | --- | --- | --- |
| **TITLE** | | |  |
| Title | 1 | Identify the report as a systematic review. | 1 |
| **ABSTRACT** | | |  |
| Abstract | 2 | See the PRISMA 2020 for Abstract checklist. | 4 |
| **INTRODUCTION** | | |  |
| Rationale | 3 | Describe the rationale for the review in the context of existing knowledge. | 7-8 |
| Objectives | 4 | Provide an explicit statement of the objective(s) or question(s) the review addresses. | 7-8 |
| **METHODS** | | |  |
| Eligibility criteria | 5 | Specify the inclusion and exclusion criteria for the review and how studies were grouped for the syntheses. | 10 |
| Information sources | 6 | Specify all databases, registers, websites, organizations, reference lists and other sources searched or consulted to identify studies. Specify the date when each source was last searched or consulted. | 9 |
| Search strategy | 7 | Present the full search strategies for all databases, registers and websites, including any filters and limits used. | 9 |
| Selection process | 8 | Specify the methods used to decide whether a study met the inclusion criteria of the review, including how many reviewers screened each record and each report retrieved, whether they worked independently, and if applicable, details of automation tools used in the process. | 9 |
| Data collection process | 9 | Specify the methods used to collect data from reports, including how many reviewers collected data from each report, whether they worked independently, any processes for obtaining or confirming data from study investigators, and if applicable, details of automation tools used in the process. | 11 |
| Data items | 10a | List and define all outcomes for which data were sought. Specify whether all results that were compatible with each outcome domain in each study were sought (e.g., for all measures, time points, analyses), and if not, the methods used to decide which results to collect. | 12 |
|  | 10b | List and define all other variables for which data were sought (e.g., participant and intervention characteristics, funding sources). Describe any assumptions made about any missing or unclear information. | 12 |
| Study risk of bias assessment | 11 | Specify the methods used to assess risk of bias in the included studies, including details of the tool(s) used, how many reviewers assessed each study and whether they worked independently, and if applicable, details of automation tools used in the process. | 11,13 |
| Effect measures | 12 | Specify for each outcome the effect measure(s) (e.g., risk ratio, mean difference) used in the synthesis or presentation of results. | 12 |
| Synthesis methods | 13a | Describe the processes used to decide which studies were eligible for each synthesis (e.g., tabulating the study intervention characteristics and comparing against the planned groups for each synthesis (item #5)). | 12 |
|  | 13b | Describe any methods required to prepare the data for presentation or synthesis, such as handling of missing summary statistics, or data conversions. | 12 |
|  | 13c | Describe any methods used to tabulate or visually display results of individual studies and syntheses. | 12 |
|  | 13d | Describe any methods used to synthesize results and provide a rationale for the choice(s). If meta-analysis was performed, describe the model(s), method(s) to identify the presence and extent of statistical heterogeneity, and software package(s) used. | 12 |
|  | 13e | Describe any methods used to explore possible causes of heterogeneity among study results (e.g., subgroup analysis, meta-regression). | 12, 15 |
|  | 13f | Describe any sensitivity analyses conducted to assess robustness of the synthesized results. | 12,15 |
| Reporting bias assessment | 14 | Describe any methods used to assess risk of bias due to missing results in a synthesis (arising from reporting biases). | 11,13 |
| Certainty assessment | 15 | Describe any methods used to assess certainty (or confidence) in the body of evidence for an outcome. | 12 |
| **RESULTS** | | |  |
| Study selection | 16a | Describe the results of the search and selection process, from the number of records identified in the search to the number of studies included in the review, ideally using a flow diagram. | 14 |
|  | 16b | Cite studies that might appear to meet the inclusion criteria but were excluded, and explain why they were excluded. | 14 |
| Study characteristics | 17 | Cite each included study and present its characteristics. | 15 |
| Risk of bias in studies | 18 | Present assessments of risk of bias for each included study. | 15 |
| Results of individual studies | 19 | For all outcomes, present, for each study: (a) summary statistics for each group (where appropriate) and (b) an effect estimate and its precision (e.g., confidence/credible interval), ideally using structured tables or plots. | 15-18 |
| Results of syntheses | 20a | For each synthesis, briefly summarize the characteristics and risk of bias among contributing studies. | 15-18 |
|  | 20b | Present results of all statistical syntheses conducted. If meta-analysis was performed, present for each the summary estimate and its precision (e.g., confidence/credible interval) and measures of statistical heterogeneity. If comparing groups, describe the direction of the effect. | 15-18 |
|  | 20c | Present results of all investigations of possible causes of heterogeneity among study results. | 15-18 |
|  | 20d | Present results of all sensitivity analyses conducted to assess the robustness of the synthesized results. | 15 |
| Reporting biases | 21 | Present assessments of risk of bias due to missing results (arising from reporting biases) for each synthesis assessed. | 15-18 |
| Certainty of evidence | 22 | Present assessments of certainty (or confidence) in the body of evidence for each outcome assessed. | 19 |
| **DISCUSSION** | | |  |
| Discussion | 23a | Provide a general interpretation of the results in the context of other evidence. | 20 |
|  | 23b | Discuss any limitations of the evidence included in the review. | 26 |
|  | 23c | Discuss any limitations of the review processes used. | 26 |
|  | 23d | Discuss implications of the results for practice, policy, and future research. | 27 |
| **OTHER INFORMATION** | | |  |
| Registration and protocol | 24a | Provide registration information for the review, including register name and registration number, or state that the review was not registered. | 26 |
|  | 24b | Indicate where the review protocol can be accessed, or state that a protocol was not prepared. | 26 |
|  | 24c | Describe and explain any amendments to information provided at registration or in the protocol. | 26 |
| Support | 25 | Describe sources of financial or nonfinancial support for the review, and the role of the funders or sponsors in the review. | 2 |
| Competing interests | 26 | Declare any competing interests of review authors. | 2 |
| Availability of data, code and other materials | 27 | Report which of the following are publicly available and where they can be found: template data collection forms; data extracted from included studies; data used for all analyses; analytic code; any other materials used in the review. | 9 |

**eTable 3:** Newcastle-Ottawa Scale Quality Assessment of Included Nonrandomized Studies

| **Study ID** | **Selection** | | | | **Comparability*** | **Outcome** | | | **Total (9**⋆**)** |
| --- | --- | --- | --- | --- | --- | --- | --- | --- | --- |
|  | Representativeness of the exposed cohort | Selection of the nonexposed cohort | Ascertainment  -nt of  exposure | Demonstration that outcome of interest was not present at start of study | Comparability of cohorts on the basis of the design or analysis | Assessment of outcome | Was follow-up  long enough  for outcomes  to occur | Adequacy of  follow-up of  cohorts |  |
| Terris, 1994 | ⋆ | ⋆ | ⋆ | ⋆ |  | ⋆ | ⋆ | ⋆ | 7 |
| Wasson, 2010 | ⋆ | ⋆ | ⋆ | ⋆ |  |  | ⋆ |  | 5 |
| Bianchi, 2011 | ⋆ | ⋆ | ⋆ | ⋆ |  | ⋆ | ⋆ | ⋆ | 7 |
| Lee, 2011 | ⋆ | ⋆ | ⋆ | ⋆ |  | ⋆ | ⋆ | ⋆ | 7 |
| Graciano, 2013 | ⋆ | ⋆ | ⋆ | ⋆ |  | ⋆ | ⋆ | ⋆ | 7 |
| Kim, 2014 | ⋆ | ⋆ | ⋆ | ⋆ |  | ⋆ | ⋆ | ⋆ | 7 |
| Zhang, 2019 | ⋆ | ⋆ | ⋆ | ⋆ |  | ⋆ | ⋆ | ⋆ | 7 |

| **eTable 4:** Summary of findings and quality assessment through the GRADE system | | | | | |
| --- | --- | --- | --- | --- | --- |
| Modified facelift incision compared with modified Blair incision for adults with presumed benign parotid tumor | | | | | |
| Patient or population: Adult patients with presumed benign parotid tumor  Setting: Adult patients with presumed benign parotid tumor  Intervention: Parotidectomy with modified facelift incision  Comparison: Parotidectomy with modified Blair incision | | | | | |
| Outcomes | Anticipated absolute effects* (95% CI) | | Relative effect (95% CI) | No. of participants  (studies) | Certainty of evidence (GRADE) |
|  | Risk with modified facelift incision | Risk with  modified Blair  incision |  |  |  |
| Cosmetic satisfaction after parotidectomy |  | Mean cosmetic satisfaction score in the intervention group was 1.66 points higher (0.89 higher to 2.46 higher) | — | 466  (4 studies) | ⨁⨁◯◯ Low^a,b,c^ |
| Operative time |  | Mean operative time in the intervention group was 0.07 hours higher (0.00 higher to 0.14 higher) | — | 73 (4 studies) | ⨁◯◯◯ Very low^a,b^ |
| Tumor size |  | Mean tumor size in the intervention group was 2.27 cm lower (4.25 lower to 0.30 lower) | — | 444 (3 studies) | ⨁◯◯◯ Very low^a,b^ |
| Incidence of temporary facial palsy | 82 per 1000  (40 to 142) | 154 per 1000 | RD -0.05 (-0.12 to 0.03) | 251 (4 studies) | ⨁◯◯◯ Very low^a,b^ |
| Incidence of permanent facial palsy | 20 per 1000  (6 to 93) | 83 per 1000 | RD -0.01 (-0.06 to 0.03) | 284 (5 studies) | ⨁◯◯◯ Very low^a,b^ |
| Incidence of Frey’s syndrome | 217 per 1000  (158 to 300) | 395 per 1000 | RD -0.18  (-0.27 to -0.10) | 365 (3 studies) | ⨁◯◯◯ Very low^a,b^ |
| Incidence of salivary complications | 64 per 1000  (57 to 71) | 61 per 1000 | RD -0.00  (-0.05 to 0.05) | 284 (5 studies) | ⨁◯◯◯ Very low^a,b^ |
| *The risk in the intervention group (and its 95% confidence interval) is based on the assumed risk in the comparison group and the relative effect of the intervention (and its 95% CI). Abbreviations: CI: confidence interval; RD: risk difference Explanations  a. Possible performance bias in all studies due to study design in nature and selection bias in two arm  b. Different types of parotidectomies are included  c. Large effect | | | | | |

**eFigure 1:** Schematic diagram illustrating the modified Blair incision (A) and the modified facelift incision (B)


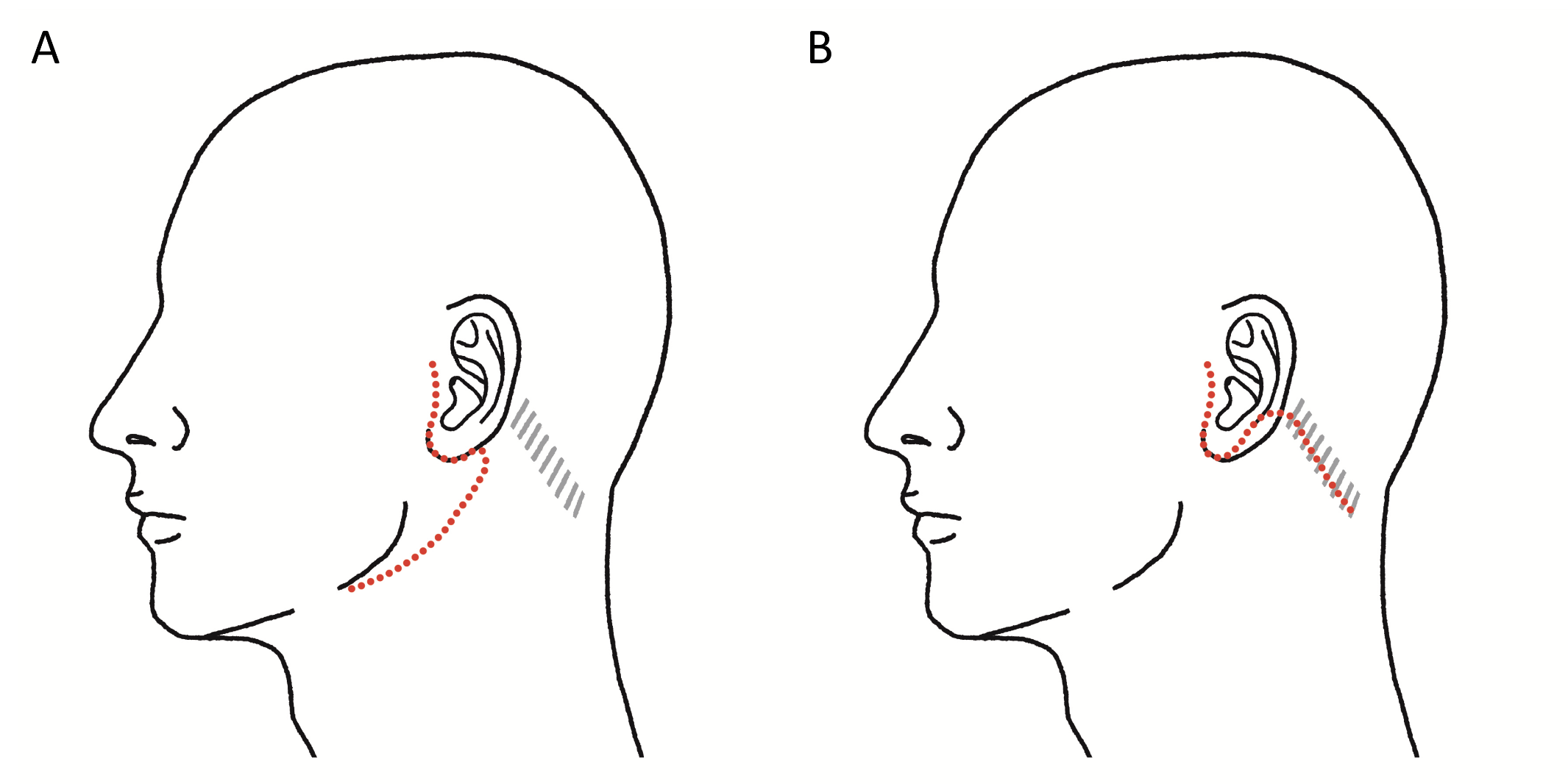

Supplement: Supplementary file 1 — Supplementary Information. [file 41598_2021_3483_MOESM1_ESM.docx]
